# Low Efficacy of Single-Dose Albendazole and Mebendazole against Hookworm and Effect on Concomitant Helminth Infection in Lao PDR

**DOI:** 10.1371/journal.pntd.0001417

**Published:** 2012-01-03

**Authors:** Phonepasong Ayé Soukhathammavong, Somphou Sayasone, Khampheng Phongluxa, Vilavanh Xayaseng, Jürg Utzinger, Penelope Vounatsou, Christoph Hatz, Kongsap Akkhavong, Jennifer Keiser, Peter Odermatt

**Affiliations:** 1 National Institute of Public Health, Ministry of Health, Vientiane, Lao People's Democratic Republic; 2 Department of Epidemiology and Public Health, Swiss Tropical and Public Health Institute, Basel, Switzerland; 3 University of Basel, Basel, Switzerland; 4 Medical Department, Swiss Tropical and Public Health Institute, Basel, Switzerland; 5 Institute of Social and Preventive Medicine, University of Zurich, Zurich, Switzerland; 6 Department of Medical Parasitology and Infection Biology, Swiss Tropical and Public Health Institute, Basel, Switzerland; McGill University, Canada

## Abstract

**Background:**

Albendazole and mebendazole are increasingly deployed for preventive chemotherapy targeting soil-transmitted helminth (STH) infections. We assessed the efficacy of single oral doses of albendazole (400 mg) and mebendazole (500 mg) for the treatment of hookworm infection in school-aged children in Lao PDR. Since *Opisthorchis viverrini* is co-endemic in our study setting, the effect of the two drugs could also be determined against this liver fluke.

**Methodology:**

We conducted a randomized, open-label, two-arm trial. In total, 200 children infected with hookworm (determined by quadruplicate Kato-Katz thick smears derived from two stool samples) were randomly assigned to albendazole (n = 100) and mebendazole (n = 100). Cure rate (CR; percentage of children who became egg-negative after treatment), and egg reduction rate (ERR; reduction in the geometric mean fecal egg count at treatment follow-up compared to baseline) at 21–23 days posttreatment were used as primary outcome measures. Adverse events were monitored 3 hours post treatment.

**Principal Findings:**

Single-dose albendazole and mebendazole resulted in CRs of 36.0% and 17.6% (odds ratio: 0.4; 95% confidence interval: 0.2–0.8; *P* = 0.01), and ERRs of 86.7% and 76.3%, respectively. In children co-infected with *O. viverrini*, albendazole and mebendazole showed low CRs (33.3% and 24.2%, respectively) and moderate ERRs (82.1% and 78.2%, respectively).

**Conclusions/Significance:**

Both albendazole and mebendazole showed disappointing CRs against hookworm, but albendazole cured infection and reduced intensity of infection with a higher efficacy than mebendazole. Single-dose administrations showed an effect against *O. viverrini*, and hence it will be interesting to monitor potential ancillary benefits of a preventive chemotherapy strategy that targets STHs in areas where opisthorchiasis is co-endemic.

**Clinical Trial Registration:**

Current Controlled Trials ISRCTN29126001

## Author Summary

Parasitic worms remain a public health problem in developing countries. Regular deworming with the drugs albendazole and mebendazole is the current global control strategy. We assessed the efficacies of a single tablet of albendazole (400 mg) and mebendazole (500 mg) against hookworm in children of southern Lao PDR. From each child, two stool samples were examined for the presence and number of hookworm eggs. Two hundred children were found to be infected. They were randomly assigned to albendazole (n = 100) or mebendazole (n = 100) treatment. Three weeks later, another two stool samples were analyzed for hookworm eggs. Thirty-two children who were given albendazole had no hookworm eggs anymore in their stool, while only 15 children who received mebendazole were found egg-negative. The total number of hookworm eggs was reduced by 85.3% in the albendazole and 74.5% in the mebendazole group. About one third of the children who were co-infected with the Asian liver fluke *Opisthorchis viverrini* were cleared from this infection following albendazole treatment and about one forth in the mebendazole group. Concluding, both albendazole and mebendazole showed disappointingly low cure rates against hookworm, with albendazole performing somewhat better. The effect of these two drugs against *O. viverrini* should be studied in greater detail.

## Introduction

Infections with the three common soil-transmitted helminths (STHs), *Ascaris lumbricoides*, *Trichuris trichiura*, and hookworm (*Ancylostoma duodenale* and *Necator americanus*), are a global public-health concern, particularly in areas where poor sanitation prevails [1], [2]. STH infections are among the most widespread of the neglected tropical diseases (NTDs) [3]. Indeed, more than a billion people are currently infected with one or several STH species, even though growing efforts are underway to control these parasitic worm infections [4]. In terms of their estimated global burden, hookworm is the most important among the STHs, perhaps responsible for more than 20 million disability-adjusted life years (DALYs) among the estimated 600 million infected people worldwide [1], [5]. Chronic hookworm infection cause intestinal blood loss and result in poor iron status and iron-deficiency anemia, particularly in children, and women in reproductive age [1], [6], [7]. As a consequence, permanent impairment, including delayed physical and cognitive development, has been described [8].

In the absence of a vaccine, the global strategy to control STHs and other NTDs is to reduce morbidity through repeated large-scale administration of anthelmintic drugs, a strategy phrased preventive chemotherapy [9]. At present, the World Health Organization (WHO) recommends four drugs against STH infections, of which albendazole and mebendazole are the two most widely used drugs for preventive chemotherapy [10]. In 2008, in the Western Pacific Region, 33.4 million children were given anthelmintic drugs [11]. According to the Lao national scheme for school deworming, there is a treatment round at the beginning of the first semester (September–December) and in the second semester (January–April). Mebendazole (single 500 mg oral dose) is annually distributed to all school-aged children since 2005 [12]. Recent efforts have been made to provide mebendazole also to preschool-aged children through the Expanded Program on Immunization (EPI) and alongside vitamin A distribution campaigns [4], [13]. However, the efficacy of mebendazole and albendazole against STH infections in Lao PDR remains to be determined, and such locally derived evidence is important to guide the national treatment policy.

The liver fluke *Opisthorchis viverrini* is co-endemic in Lao PDR, and particularly high prevalences have been observed in the southern provinces [14]–[17]. Praziquantel is the current drug of choice against *O. viverrini* [3]. Previous work has shown that multiple doses of albendazole also show some effect [18], [19]. Hence, in areas where STHs and *O. viverrini* co-exist and preventive chemotherapy targeting STHs is under way, it will be interesting to monitor for potential ancillary benefits of this control strategy against opisthorchiasis.

The purpose of this study was to assess the efficacy of single-dose albendazole (400 mg) and single-dose mebendazole (500 mg) against hookworm infection among school-aged children in Lao PDR. In addition, the effect on other STHs (i.e., *A. lumbricoides* and *T. trichiura*) and *O. viverrini* in co-infected children was assessed. Our study complements a recent investigation, done in the People's Republic of China that compared single and triple dosing with albendazole and mebendazole against the three common STHs [20].

## Methods

### Ethics Statement

The research protocol (see Protocol S1) was approved by the Ethics Committee of Basel, Switzerland (EKBB; reference no. 146/08) and the Lao National Ethics Committee for Health Research (NECHR), Ministry of Health, Vientiane, Lao PDR (reference no. 170/NECHR). The trial is registered with Current Controlled Trials (identifier: ISRCTN29126001). Written informed consent was obtained from parents/legal guardians of eligible children. Participation was voluntary and children could withdraw from the trial at any time without further obligation.

At completion of the trial, all children of the two primary schools and participants who were still found positive for hookworm (or other STHs) were treated with albendazole (400 mg). *O. viverrini-*infected children were administered praziquantel according to national guidelines [21].

### Study Area and Population

A randomized, open-label trial was carried out between February and March 2009 in two primary schools (Oudomsouk and Nongbok Noi) in Batieng district, Champasack province, southern Lao PDR. Children in the two schools were treated with mebendazole 5–6 months prior to the start of our study. The schools are located approximately 15 km southeast of Pakse town, on the Bolaven plateau at an altitude of approximately 1,000 m above sea level (geographical coordinates: 105°56′53″N latitude, 15°14′59″E longitude). The rainy season lasts from May to mid-October. A census done in 2007 revealed that 43,651 people lived in the 95 villages of Batieng district (Dr. Nanthasane Vannavong, Champasack Provincial Health Department; personal communication). More than three-quarter of the households (77.5%) lack appropriate sanitation. Drinking water is primarily obtained from unprotected boreholes and wells. Most villagers live on subsistence rice farming and rubber plantations (Dr. Nanthasane Vannavong, Champasak Provincial Health Department; personal communication). Infections with STHs and *O. viverrini* are common in Batieng district; a recent study revealed infection prevalences above 50% and above 20%, respectively [22].

### Study Design

We designed a randomized, open-label trial comparing albendazole (single 400 mg dose) and mebendazole (single 500 mg dose) for treatment of hookworm infection. The sample size was calculated based on results of a meta-analysis on the efficacy of current anthelmintic drugs against common STH infections, which reported cure rates (CR; defined as percentage of helminth-positive individuals who became helminth-egg negative after treatment) of 75% and 15% for albendazole (400 mg) and mebendazole (500 mg), respectively against hookworm infection [10]. In order to account for the large variation (uncertainty) of the observed efficacy of mebendazole in the individual studies (CRs of 8–91% were found in the six randomized controlled trials), we more than tripled the mean efficacy of mebendazole (50% instead of 15%). Assuming superiority of albendazole (1-tailed test) and taking into account a 90% power, and an alpha error of 5%, we obtained a sample size of 85 children per treatment group. Furthermore, we assumed a drop-out rate of 15%, which resulted in a total sample size of 200 hookworm-positive school-aged children.

### Field and Laboratory Procedures

The teachers of the two primary schools, the children, and the staff of the National Institute of Public Health, Centre of Malaria, Parasitology and Entomology, Centre for Laboratory and Epidemiology, the Provincial Department of Health, the Provincial Hospital, and the Malaria Station of Champassak, and the village authorities were informed one week in advance on the study aims and procedures. Potential risks and benefits were explained to all children and their parents/guardians. An informed consent form was distributed to all parents/guardians and signed. Children assented orally.

At baseline screening the consenting children (n = 465) of the two schools, aged 6–12 years, provided two fresh stool samples within a period of 3 days. Stool containers were filled by children and transferred to a laboratory in the early morning (between 8 and 9 am). All collected specimens were worked up on the day of collection. From each stool sample, duplicate Kato-Katz thick smears were prepared on microscope slides, using standard 41.7 mg templates [23]. Kato-Katz thick smears were quantitatively examined under a light microscope for helminths with a 100× magnification. Slides were read within 30–45 min after preparation. A random sample of approximately 10% of the Kato-Katz thick smears were re-examined by two senior technicians for quality control purposes. In case of discrepancies (i.e., positive *vs.* negative results and egg counts differing by >10%), results were discussed with the respective technicians, and the slides re-examined until agreement was reached.

In addition, a questionnaire was administered to each participating child to obtain sociodemographic data (i.e., sex, age, parent's education and occupation, ethnic group, and sanitation infrastructure), and behavioral data (i.e., wearing shoes, sources of drinking water, food consumption, and personal hygiene). Hookworm-positive children (defined by the presence of at least one hookworm egg in one of the quadruplicate Kato-Katz thick smears examined per child) were invited for treatment (n = 200).

At enrollment, a clinical examination, which included measurement of weight (using an electronic balance measured to the nearest 0.1 kg), height (using a measuring tap fixed to the wall and measured to the nearest cm), and axcillary temperature (using battery-powered thermometers, measured to the nearest 0.01°C), anemia assessment (finger prick capillary blood sample) was conducted, and a medical history taken. Children were excluded if they had fever, or showed signs of severe malnutrition. Additional exclusion criteria were the presence of any abnormal medical condition such as cardiac, vascular, pulmonary, gastrointestinal, endocrine, neurologic, hematologic (e.g., thalassaemia), rheumatologic, psychiatric, or metabolic disturbances, recent history of anthelmintic treatment (e.g., albendazole, mebendazole, pyrantel pamoate, levamisole, ivermectin, and praziquantel), attending other clinical trials during the study, or reported hypersensitivity to albendazole or mebendazole.

At follow-up, 21–23 days after drug administration, two stool samples were collected from each child and transferred to a hospital laboratory within one hour after collection. Each stool specimen collected at follow-up was subjected to the same procedures as during the baseline survey. Hence, duplicate Kato-Katz thick smears were prepared from each stool sample, examined under a microscope within 30–45 min by experienced laboratory technicians, and helminth eggs were counted and recorded for each species separately. We adhered to the same quality control as during the baseline survey.

### Randomization

Children were randomly assigned to a single dose of albendazole (400 mg) or mebendazole (500 mg), using a block randomization procedure (six blocks each containing four treatment allocations), generated by an independent statistician who was not otherwise involved in the trial. The sequence of blocks was determined using a random number table. In addition, schools were decoded by a researcher to assign children either to albendazole or mebendazole. Eligible children were randomly assigned and allocated to treatment by an epidemiologist. Children and drug administrators were not blinded for drug treatment. Laboratory personnel and clinicians monitoring the adverse events were blinded throughout the study.

### Drugs and Adverse Events

Albendazole (400 mg; Albendazole®, South Korea) was obtained from the Ministry of Health, Vientiane, Lao PDR. Mebendazole (500 mg; Vermox®, Italy) was donated by Johnson & Johnson Pharmaceuticals, provided through the Ministry of Health and the Ministry of Education, Vientiane, Lao PDR. At treatment day, both groups received the drugs under direct medical supervision on an empty stomach. Children were monitored for at least 3 hours after drug administration and asked to report for any drug-related adverse events using a standard questionnaire administered and graded by study physicians.

### Statistical Analysis

Data were double-entered and cross-checked in EpiData version 3.1 (EpiData Association; Odense, Denmark). Statistical analyses were performed with STATA, version 10.1 (Stata Corp.; College Station, TX, USA). Efficacy was calculated for both intention-to-treat (ITT) and per-protocol (PP) analyses. ITT analysis was based on the initial treatment intent. PP analysis included only those children who had complete data records (i.e., quadruplicate Kato-Katz thick smear reading before and after treatment, and full treatment compliance).

Infections with hookworm, *A. lumbricoides*, *T. trichiura*, and *O. viverrini* were grouped into light, moderate, and heavy infections, according to WHO guidelines (for STHs) and cut-offs put forward by Maleewong and colleagues and WHO (for *O. viverrini*) [24], [25]. Infection intensity classifications are as follows: hookworm, 1–1,999 eggs per gram of stool (EPG) (light), 2,000–3,999 EPG (moderate), and ≥4,000 EPG (heavy); *A. lumbricoides*, 1–4,999 EPG (light), 5,000–49,999 EPG (moderate), and ≥50,000 EPG (heavy); and *T. trichiura* and *O. viverrini*, 1–999 EPG (light), 1,000–9,999 EPG (moderate), and ≥10,000 EPG (heavy).

Primary outcome measures were CR and egg reduction rate (ERR), the latter defined as the positive group's reduction of geometric mean (GM) fecal egg count at posttreatment, divided by the GM fecal egg count at pretreatment, multiplied by 100. Additionally, changes in class of infection intensities were determined following treatment. Negative binomial regression was applied to compare ERRs observed between both treatment groups. A Wilcoxen test was employed for the matched pair's analysis. We determined egg reduction rate ratio (ERRR) and 95% confidence interval (CI). Pearson's χ^2^-test and Fisher's exact test, as appropriate, were used to assess the baseline binary characteristics between the treatment arms. Statistical significance was estimated using a likelihood ratio test (LRT). *P*-value below 5% was considered significant.

CONSORT checklist was followed to report on the trial (see Checklist S1).

## Results

### Study Cohort

**Figure 1:**
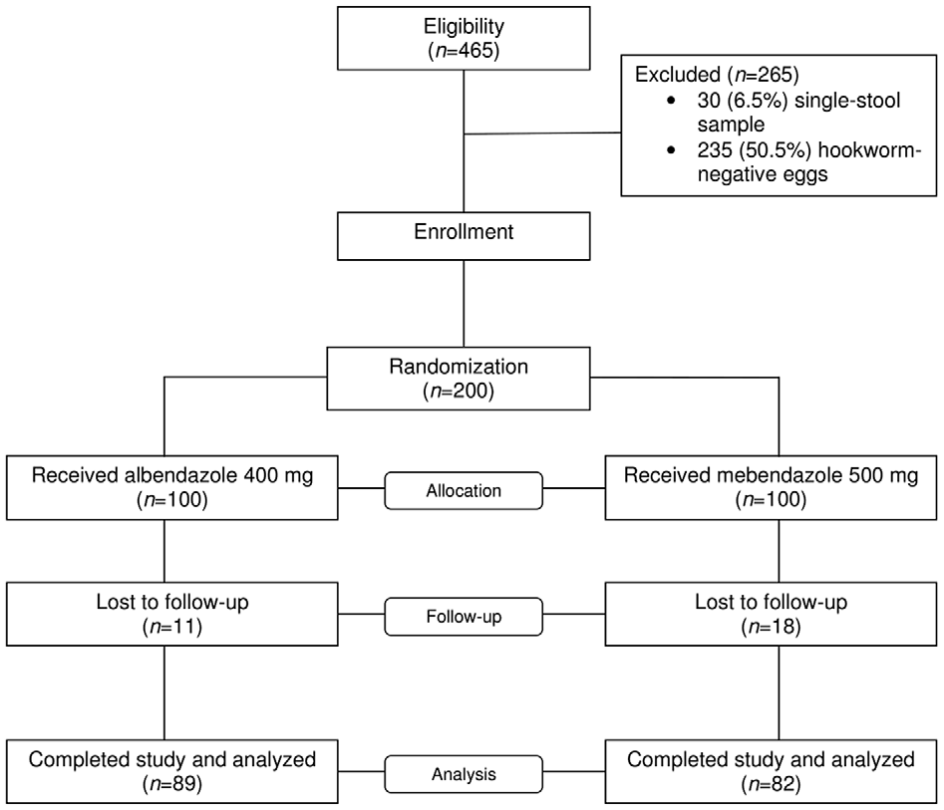
Flow chart detailing the study participation and compliance. Children who completed two stool samples were included in the final analysis for assessing the efficacy of single-dose albendazole (400 mg) and single-dose mebendazole (500 mg) treatment against hookworm and concomitant helminth infections in Bachieng district, Champasack province, southern Lao PDR in February/March 2009.

Four hundred sixty-five school-aged children were enrolled in the baseline screening. Two hundred children (43.0%), 130 boys and 70 girls with a parasitologically confirmed hookworm infection, were randomly assigned to one of the two treatments. Data of these 200 children were included in the ITT analysis. The remaining 265 children were excluded because they had no hookworm eggs in their stool (n = 235) or provided only a single stool sample (n = 30). Overall, 171 children (85.5%) had complete baseline data, received treatment, and completed follow-up examinations, and hence PP analysis was performed on these children. Twenty-nine children (14.5%) were lost to follow-up, 18 in the mebendazole and 11 in the albendazole group (Figure 1). The 171 children with complete data records were included in the primary analysis. Their parents most commonly had completed primary school only (77.5% of parents for the albendazole group and 80.5% for the mebendazole group). The most common profession of patients' parents was farming with 49.4% and 62.2% for albendazole and mebendazole treatment groups, respectively. The two groups were similar in terms of household assets, source of drinking water and consumption of cooked foods as well as raw fish (data not shown). More specifically, the consumption of raw fish was reported by 61.8% and 58.5%, respectively, and included dishes like “Pa Dek” (fermented fish sauce), “Lap Pa”, and “Koy Pa” (raw, fish-based dishes).

### Baseline Characteristics

At baseline, characteristics of the two treatment groups were similar (Table 1), including age (albendazole recipients: mean (standard deviation, SD) age 8.4 (2.1) years; mebendazole recipients: 8.7 (2.1) years), weight (mean (SD) 23.8 (5.8) kg and 25.0 (5.9) kg, respectively), height (mean (SD) 123.8 (11.0) cm and 126.9 (11.0) cm, respectively), and hemoglobin (Hb) concentration (mean (SD) 11.8 (1.1) mg/dl and 11.9 (1.3) mg/dl, respectively). In both treatment groups, most children were diagnosed with a light hookworm infection (82.0%), whereas the remaining children had moderate or heavy infection intensities. The hookworm GM fecal egg counts in the mebendazole and albendazole groups were 707.0 and 859.1 EPG, respectively (Table 2).

**Table 1:** Baseline characteristics of 171 hookworm-infected school children, Bachieng district, Champasak province, Lao PDR, in February/March 2009.

	Albendazole(*n* = 89)	*Mebendazole* *(n = 82)*
Boys/girls	56/33	*49/33*
Mean (SD) age, years	9.0 (2.1)	*9.0 (2.1)*
Mean (SD) weight, kg	24.0 (6.0)	*25.2 (6.0)*
Mean (SD) height, cm	124.1 (11.0)	*127.0 (11.0)*
Mean (SD) hemoglobin, mg/dl	11.9 (1.1)	*12.4 (1.3)*
Anemia (<11.5 mg/dl), n, (%)^a^	23 (57.5)	*17 (42.5)*
Latrine facility present, n, (%)	5 (5.6)	*1 (1.2)*
Parasitic infections		
Hookworm infection^b^		
Light (1–1,999 EPG)	72 (80.9)	*67 (81.7)*
Moderate (2,000–3,999 EPG)	9 (10.1)	*7 (8.6)*
Heavy (≥4,000 EPG)	8 (9.0)	*8 (9.7)*
Co-infection with		
*Ascaris lumbricoides* b		
Negative	61 (68.5)	*53 (64.6)*
Light (1–4,999 EPG)	18 (20.2)	*18 (22.0)*
Moderate (5,000–49,999 EPG)	7 (7.9)	*8 (9.8)*
Heavy (≥50,000 EPG)	3 (3.4)	*3 (3.7)*
*Trichuris trichiura* b		
Negative	51 (57.3)	*39 (47.6)*
Light (1–999 EPG)	38 (42.7)	*43 (52.4)*
Moderate (1,000–9,999 EPG)	4 (4.5)	*0*
Heavy (≥10,000 EPG)	0	*0*
*Taenia* spp.		
Negative	78 (87.6)	*79 (96.3)*
Positive	11 (12.4)	*3 (3.7)*
*Opisthorchis viverrini* c		
Negative	44 (49.4)	*50 (61.0)*
Light (1–999 EPG)	41 (46.1)	*25 (30.5)*
Moderate (1,000–9,999 EPG)	4 (4.5)	*7 (8.5)*
Heavy (≥10,000 EPG	0	*0*

a *According to guidelines put forth by WHO regarding definition of anemia* [*42*].

b *According to guidelines put forth by WHO* [*25*] *, based on Kato-Katz thick smear examination*.

c *According to Maleewong and colleagues* [*24*] *, based on Kato-Katz thick smear examination*.

*Data are no; (%) of subject, otherwise indicated (95% confidence interval); EPG, eggs per gram of stool; GM, geometric mean*.

**Table 2:** Hookworm infection at baseline and follow-up and cure rate of albendazole and mebendazole (per-protocol analysis).

	Pretreatment		*Posttreatment*	
	Albendazole(*n* = 89)	Mebendazole(*n* = 82)	Albendazole(*n* = 89)	*Mebendazole* *(n = 82)*
No. of hookworm-infected patients	89 (100)	82 (100)	57 (64.0)	*67 (81.7)*
No. of children cured (cure rate, %)	n.a.	n.a.	32 (36.0)	*15 (17.6)* a
Light infection (1–1,999 EPG)	72 (80.9)	67 (48.2)	55 (61.8)	*59 (72)*
No. of children cured (cure rate, %)	n.a.	n.a.	17 (19.1)	*8 (9.8)* b
Moderate infection (2,000–3,999 EPG)	9 (18.0)	7 (46.7)	2 (2.2)	*6 (7.3)*
No. of children cured (cure rate, %)	n.a.	n.a.	7 (7.9)	*1 (1.2)* c
Heavy infection (≥4,000 EPG)	8 (1.1)	8 (1.1)	0 (0)	*2 (2.4)*
No. of children cured (cure rate, %)	n.a.	n.a.	8 (9)	*6 (7.3)* d
GM fecal egg count (range), EPG	859.1 (699.0–1,057.0)	707.0 (559.0–894.3)	63.0 (34.0–116.0)	*147.3 (90.0–242.0)*
Egg reduction rate, %	n.a.	n.a.	86.7	*76.3* e

a *OR 0.4 [95% CI (0.2–0.8; P = 0.01)] comparison of treatment outcomes between mebendazole vs. albendazole;*

b *P = 0.13;*

c *P = 0.04;*

d *P = 0.46;*

e *ERRR 1.0 [95% CI (0.7–1.6; P = 0.90)] comparison of treatment outcomes between mebendazole vs. albendazole*.

*Note. Data are number; (%) of children, unless otherwise indicated (95% confident interval); GM, geometric mean; EPG, eggs per gram of stool; ERRR egg reduction rate ratio; OR odds ratio; n.a. not applicable*.

The overall infection rates of *A. lumbrico*i*des, O. viverrini* and *T. trichiura* were 34.0%, 48% and 45.0%, respectively. *O. viverrini* GM fecal egg counts were 84.9 EPG (albendazole) and 120.8 EPG (mebendazole) (Table 3).

**Table 3:** Infection rate and cure rate of albendazole and mebendazole for hookworm co-infections.

	Pretreatment		*Posttreatment*	
	Albendazole	Mebendazole	Albendazole	*Mebendazole*
Parasitic infection				
*A. lumbricoides* (n = 58)	(*n* = 28)	(*n* = 30)	(*n* = 28)	*(n = 30)*
No. of *A. lumbricoides-*infected children	28 (100)	30 (100)	2 (7.1)	*2 (6.7)*
No. of patients cured (cure rate, %)	n.a.	n.a.	26 (92.9)^a^	*28 (93.3)* a
GM fecal egg count (range), EPG	1,567.0 (553.0–4,444.0)	1,584.0 (528.0–4,751.0)	0	*0*
ERR, %	n.a.	n.a.	100^b^	*100* b
*T. trichiura* (n = 82)	(*n* = 39)	(*n* = 43)	(*n* = 39)	*(n = 43)*
No. of *T. trichuris-*infected children	39 (100)	43 (100)	26 (66.7)	*31 (72.1)*
No. of patients cured (cure rate, %)	n.a.	n.a.	13 (33.3)	*12 (27.9)* c
GM fecal egg count (range), EPG	94.1 (48.3–184.0)	65.2 (39.3–108.3)	75.0 (42.2–133.2)	*48.0 (25.0–93.0)*
ERR	n.a.	n.a.	67.0^d^	*66.0* d
*O. viverrini* (n = 77)	(*n* = 45)	(*n* = 32)	(*n* = 45)	*(n = 32)*
No. of *O. viverrini-*infected children	45 (100)	32 (100)	30 (66.7)	*25 (75.8)*
No. of patients cured (cure rate, %)	n.a.	n.a.	15 (33.3)^e^	*8 (24.2)* e
GM fecal egg count (range), EPG	84.9 (41.8–184.0)	120.8 (48.9–297.9)	73.0 (34.3–155.7)	*114.4 (48.9–267.3)*
ERR, %	n.a.	n.a.	82.1^f^	*78.2* f

a *OR 0.8 [95% CI (0.2–2.6; P = 0.71) comparison of treatment outcomes between mebendazole vs. albendazole*.

b *ERRR n.a.*

c *OR 0.8 [95% CI (0.3–1.9; P = 0.58)] comparison of treatment outcomes between mebendazole vs. albendazole*.

d *ERRR 0.7 [95% CI (0.3–1.2; P = 0.22)] comparison of treatment outcomes between mebendazole vs. albendazole*.

e *OR 0.7 [95% CI (0.3–1.9; P = 0.62)] comparison of treatment outcomes between mebendazole vs. albendazole*.

f *ERRR 0.8 [95% CI (0.2–3.9; P = 0.78)] comparison of treatment outcomes between mebendazole vs. albendazole*.

*Note. Data are number; (%) of children, unless otherwise indicated (95% confident interval); GM, geometric mean; EPG, eggs per gram of stool; ERRR, egg reduction rate ratio; OR odds ratio; n.a. not applicable*.

### Albendazole and Mebendazole Efficacy against Hookworm

In the ITT analysis, the CRs of albendazole and mebendazole against hookworm infection were 32.0% and 15.0%, respectively. Overall, 124 children (73%) remained hookworm-egg positive; 68 receiving albendazole and 85 in the mebendazole treatment group. Similar results were obtained with the PP analysis (Table 2). A statistically significant difference was observed when comparing the observed CRs using albendazole *vs.* mebendazole (OR = 0.4; 95% CI 0.2–0.8; *P* = 0.01). The hookworm GM fecal egg counts obtained at follow-up were 63.0 EPG in albendazole recipients and 147.3 EPG in mebendazole recipients (ITT analysis 96.5 EPG and 210 EPG, respectively). The respective ERRs for albendazole and mebendazole were 86.7% and 76.3% (ERRR 1.0; 95%CI 0.7–1.6; *P* = 0.90. In children with moderate infection intensities (2,000–3,999 EPG), the effect of albendazole and mebendazole was significantly different (*P* = 0.04).

### Effect of Albendazole and Mebendazole against *A. lumbricoides, T. trichiura*, and *O. viverrini*

Table 3 shows the effect of albendazole and mebendazole against *A. lumbricoides*, *T. trichiura*, and *O. viverrini*. At baseline, GM infection intensities of *A. lumbricoides* were 1,567 EPG in albendazole recipients and 1,584 EPG in mebendazole recipients. Both albendazole and mebendazole treatments achieved high CRs above 90% and resulted in almost complete egg elimination. The CRs of albendazole and mebendazole obtained against *T. trichiura* were 33.3% and 27.9%, respectively. The respective ERRs were 67.0% and 66.0%. No statistically significant difference was observed for CR and ERR between the two treatments (OR = 0.8; 95% CI 0.3–1.9; *P* = 0.58 and ERRR = 0.7; 95% CI 0.3–1.2, *P* = 0.22). Finally, CRs against *O. viverrini* achieved with albendazole and mebendazole were 33.3% and 24.2%, respectively (OR = 0.7; 95% CI 0.3–1.9; *P* = 0.62). The respective ERRs were 82.1% and 78.2% (ERRR = 0.8; 95% CI 0.2–3.9, *P* = 0.78).

### Adverse Events

Monitoring of children within 3 hours after drug administration revealed no drug-related adverse events, neither in the albendazole nor in the mebendazole group. Hence, both treatments were well tolerated.

## Discussion

This current head-to-head comparison of single-dose albendazole *vs.* mebendazole against hookworm infection in Lao school-aged children – to our knowledge the first comparative trial in this Southeast Asian country – shows sobering results. Indeed, the standard single oral doses of albendazole (400 mg) and mebendazole (500 mg) that are recommended for preventive chemotherapy targeting STHs [8], [9] resulted in low CRs against hookworm infection (36.0% and 17.6%, respectively). The respective ERRs were moderate, (86.7% and 76.3%).

A sizeable number of children were co-infected with *A. lumbricoides*, *T. trichiura*, and *O. viverrini*, which allowed us to determine the effect of albendazole and mebendazole against these helminth species. With regard to *A. lumbricoides*, high efficacy of both drugs was confirmed against this helminth species [3], [10]. Our study also confirms the previously reported low efficacy of both drugs against *T. trichiura* [3], [10], [26].

While the results obtained with mebendazole against hookworm and the efficacy observed with both drugs against *A. lumbricoides* and *T. trichiura* are in line with previous studies [20], [27], [28] and in agreement with overall CRs estimated through a meta-analysis [10], the low CR (36.0%) achieved with albendazole in the treatment of hookworm infection is somewhat surprising. Indeed, in the aforementioned meta-analysis, randomized controlled trials of single-dose albendazole (400 mg) revealed an overall CR against hookworm of 75% [10]. The reasons for the considerably lower efficacy of albendazole observed in our study are unclear. Quality control of drug samples performed in our laboratories revealed that disintegration, dissolution, and concentration of the albendazole tablets used in our trial were comparable to Zentel® (data not shown). The hookworm species (and strains) endemic in southern Lao PDR might be an explanation. However, there is a paucity of information on which hookworm species is predominant in Southeast Asia. Indeed, in our study setting the infection rates of the two hookworm species, *A. duodenale* and *N. americanus*, are not known. Furthermore, recent studies documented that in Southeast Asia humans are at risk of acquiring *Ancylostoma ceylanicum*, which is endemic in dogs and cats of the region and its importance in humans might be underestimated [29], [30]. Hence, further analysis on the circulating parasite species is required to elucidate this issue. In addition, day-to-day variability in hookworm egg counts from individuals is a well described phenomenon [31]. Finally, the study's sample size is rather small and therefore a few incidental effects such as failure of some children to swallow the tablet correctly, might have contributed to low efficacy of albendazole for the treatment of hookworm infection. To sum up, differences in strain and species susceptibilities, host factors, and co-infections with other helminths are factors that might all play a role in explaining treatment failures [28], [32].

Nevertheless, we cannot rule out that albendazole resistance is developing in our study setting. To date, nematode resistance in humans has not been reported. On the other hand, drug resistance is a major problem in veterinary public health [33], [34]. The development of broad spectrum anthelmintic resistance, in particular resistance of nematodes to benzimidazoles, has been recognized in ruminants for decades [34], [35]. Extensive studies on the underlying mechanisms of drug resistance have been carried out [36]. Further investigations on failure of the drugs to completely cure the patients are necessary in our study setting to substantiate this suspicion.

It is interesting to note that the two drugs employed, even at single oral doses, showed some effect against *O. viverrini*. Although CRs were low (24.2–33.3%), the moderate ERRs of 78.2–82.1% are encouraging. At present, praziquantel is the drug of choice against opisthorchiasis [3], [18]. Studies carried out in the 1980s in *O. viverrini*-infected hamsters and patients infected with *O. viverrini* documented opisthorchicidal properties of albendazole and mebendazole [19], [37]. However, long treatment courses of up to 7 days were recommended in view of these initial laboratory and clinical findings. Experiences with long treatment courses have been reported from a hospital-based randomized trial; albendazole given at dosages of 400 mg twice daily for 3 and 7 days resulted in CRs of 40% and 63%, respectively, and corresponding ERRs of 92% [19]. Furthermore, mebendazole in dosages of 30 mg/kg daily for 3 or 4 weeks resulted in CRs of 94% against *O. viverrini*. Long treatment courses compromise compliance, increase costs and are not feasible for large-scale community-based control, which might explain that albendazole and mebendazole were not further promoted for *O. viverrini* treatment [37].

It should be noted that in our study Kato-Katz thick smears served as method for helminth diagnosis. However, this diagnostic approach does not allow differentiating the eggs of *O. viverrini* from minute intestinal flukes [38], [39]. In addition, since the emphasis of our research was on hookworm, the efficacy of albendazole and mebendazole against other STHs and *O. viverrini* could not be compared with the appropriate sample sizes. Finally, mostly light *O. viverrini* infections were present in our study and the sample of *O. viverrini*-infected patients was not representative of the overall community as hookworm infection was the leading selection criterion. Hence, additional clinical investigations are warranted to assess the opisthorchicidal properties of albendazole and mebendazole in comparison to praziquantel. Moreover, the anthelmintic drug tribendimidine [40] showed high CR and ERR against *O. viverrini* in a recent, open-label exploratory trial carried out in Lao PDR [41]. It would therefore be interesting to conduct a four-arm study, comparing praziquantel (treatment of choice) with tribendimidine, albendazole, and mebendazole.

In conclusion, we have assessed the efficacy of standard single-dose regimens of albendazole and mebendazole against hookworm infection in school-aged children from Lao PDR and provide further evidence of the effects these two drugs have against other helminth species concurrently harbored in the human host. Both drugs showed a similar profile, with low efficacy against hookworm and, additionally, low efficacy against *T. trichiura*, and high efficacy against *A. lumbricoides*. The low efficacy of single-dose of albendazole against hookworm should be followed-up closely and further investigated as this drug is widely used for preventive chemotherapy against STHs and in combination with ivermectin in the current global effort to eliminate lymphatic filariasis. The effects of the two drugs against *O. viverrini* warrant further investigations, in comparison with the current drug of choice praziquantel as well as tribendimidine.

## Supporting Information

Checklist S1 **CONSORT Checklist.**
(DOC)

Protocol S1 **Trial Protocol.**
(PDF)
