# Supplementary material for: Low Efficacy of Single-Dose Albendazole and Mebendazole against Hookworm and Effect on Concomitant Helminth Infection in Lao PDR
Source: PLoS Negl Trop Dis. 2012 Jan 3;6(1):e1417. doi: 10.1371/journal.pntd.0001417 (PMC3250499; doi:10.1371/journal.pntd.0001417)

Title	**Comparative efficacy of albendazole and mebendazole against hookworm infection: randomized, controlled trial in schoolchildren in Khamkeuth district, Bolikhamxay province, Lao PDR**
Draft – Version	3 April 2008
Study site	Selected primary schools in Batieng district, Champasack province, in southern Lao PDR
Study period	Baseline period from 28 April to 4 May 2008<br>Follow up period from 27 to 30 May 2008
Research institutions and investigators	**National Institute of Public Health (Vientiane, Lao PDR):** Phonepasong Soukhathammavong, Somphou Sayasone, Khampheng Phongluxa, Monly Vanmany<br>**Swiss Tropical Institute (Basel, Switzerland):** Peter Odermatt, Christoph Hatz, Jennifer Keiser, Jürg Utzinger

CONTENTS

1. Objective

2. Background

3. Hypotheses

4. Methods

    4.1 Study design

    4.2 Intervention

    4.3 Primary and secondary outcomes

    4.4 Study area and population

    4.5 Time schedule

    4.6 Sample size calculation

    4.7 Outcome measurements

    4.8 Study procedures

    4.9 Analysis

5. Ethical considerations

6. References

Appendices:

    Appendix 1: Step-by-step field procedures

    Appendix 2: Flow chart for the work in the field site

1. OBJECTIVE

To compare the efficacy of oral albendazole (single 400-mg dose) and oral mebendazole (single 500-mg dose) against hookworm infection among schoolchildren in central Lao PDR.

2. BACKGROUND

Soil-transmitted helminthiases, especially intestinal nematode infections (i.e. *Ascaris lumbricoides*, hookworm and *Trichuris trichiura*), constitute a major public health problem worldwide. However, the bulk of the burden due to soil-transmitted helminthiases is concentrated in rural areas and deprived urban settings of low-income countries where clean water and improved sanitation are lacking and access to health services and treatment is inadequate. It is currently estimated that globally more than half a billion individuals are infected with hookworms (*Ancylostoma duodenale* and *Necator americanus*), the majority of whom are children (de Silva et al., 2003; Bethony et al., 2006). As many as 65,000 individuals might die each year due to the long-term complications of chronic hookworm disease (WHO, 2002; Hotez et al., 2006a). Moreover, a hookworm infection is considered as the second most important parasitic infection among the so-called neglected tropical diseases, according to the disability-adjusted life year (DALY) metrics (Hotez et al., 2006a).

There is no vaccine for the prevention of hookworm disease (Hotez et al., 2006b). Morbidity control is the current mainstay of control of hookworm disease and preventive chemotherapy has been endorsed by the World Health Assembly in May 2001 (Utzinger & Keiser 2004; Bethony et al., 2006). At present, there are 4 drugs on the WHO model list of essential medicines, i.e. albendazole, mebendazole, levamisole and pyrantel pamoate. The former two belong to the benzimidazole carbamates, which are the most widely used anthelminthics in large-scale control programmes due to their low cost, ease of administration and good safety and therapeutic profiles.

High hookworm prevalence rates have been reported from rural parts of Lao PDR. For example, in a recent study carried out in Champasak province, the prevalence of hookworm infection was above 85%. Particularly affected are Ban Pcew in Mounlapamok district, Ban Huay Chod, Ban Namtuat and Nonphanuan in Paksong district, and Ban Lon Song in Khong district (Somphou Sayasone 2006-2007 & Tippi Mak 2006 unpublished reports). In the entire country, school deworming has started (usually carried out twice per year). Biannual treatment with mebendazole (single 500-mg oral dose) is a pillar of the control activities.

Low efficacies of mebendazole against hookworm infections have been reported from Mali, Tanzania and Vietnam (De Clercq et al., 1997, Albonico et al., 2003; Flohr et al. 2007). Albendazole, on the other hand, resulted in high cure rates against hookworm infections (for a review see: Horton, 2000). In view of the low cure rates obtained with mebendazole against hookworm infections, there is a pressing need to conduct well-designed trials that provide new data regarding the efficacy of commonly used anthelminthics against hookworm infections in different settings, so that the most efficacious drugs can be used in control programmes.

3. HYPOTHESES

Mebendazole (MBZ) 500-mg, single oral dose, has a significantly inferior efficacy to cure (complete clearance, π) and reduce intensity of infection (mean eggs per gram [epg] stool) rate for treatment of hookworm infections compared with albendazole (ABZ) 400-mg, single oral dose:

$Ho = \varphi\ MBZ - \varphi\ ABZ > \delta$; and
$Ha = \varphi\ MBZ - \varphi\ ABZ < \delta$ (δ is the minimum difference of clinical significance)

4. METHODS

4.1 Study design: Randomized, controlled trial in schoolchildren.

4.2 Intervention: Children will be randomly assigned to receive either MBZ 500-mg (Group A) or ABZ 400-mg (Group B). Drugs will be administered at a single oral dose.

4.3 Primary outcome measures

1. Hookworm parasite clearance (no egg excretion of initially hookworm-positive children after treatment), assessed 21-23 days post-treatment.
2. Hookworm egg reduction rate (reduction of hookworm intensity, measured in epg) 21-23 days post-treatment.

4.4 Study area and population

1. One selected primary school in a village with a known high prevalence of hookworm among schoolchildren (≥50%) in Batieng district, Champasack province, in southern Lao PDR.
2. Schoolchildren, aged 5-16 years, who appear healthy upon a general clinical check, will be eligible.

4.5 Time schedule

1. Baseline screening (from 28nd to 4th May 2008): From each child two stool samples will be collected within 2-3 days and examined with the Kato-Katz method (Katz et al., 1972). Two Kato-Katz thick smears will be prepared from each stool sample. Hookworm-positive children (at least 1 hookworm egg in one of the four Kato-Katz thick smears) will be treated.

2. End-of-study screening (from 27th to 30th May 2008; 21-23 days post-treatment): From each child, 2 stool samples will be obtained within 2-3 days and duplicate Kato-Katz thick smears will be examined as before.

4.6 Sample size

In a recent meta-analysis carried out at the Swiss Tropical Institute on the efficacy of the 4 anthelminthics that are on the WHO model list of essential drugs, the efficacy (parasite clearance rates, cure rate) of MBZ (500-mg single dose) and ABZ (400-mg single dose) was estimated at 15% (6 randomized, placebo-controlled trials, range 8-91%) and 75% (14 randomized, placebo controlled trials; range 45-100%), respectively (Keiser & Utzinger, 2008). The efficacy of mebendazole varied largely. In order to account for this variation (uncertainty) we tripled the mean efficacy for the sample size calculations (51% instead of 15%). The estimated ALB efficacy of 75% was compared with 51% for MBZ. With a 90% power, and an alpha error of 5%, we calculated a sample of 85 children per treatment group. (Calculation assumes superiority of ABZ: 1- tailed test, Table 1). In total 170 children are required. Given a drop-out rate of 10% we need to enroll 190 children with a hookworm infection. Assuming a hookworm prevalence of 50% in the study area, approximately 400 children will need to be screened to identify the required number of study participants. Taking into account that from each individual 2 stool samples will be examined 800 stool samples will need to be examined in the base-line survey resulting in 1,600 Kato-Katz thick smears. In the follow-up examination 21-23 days after treatment, only the treated children (i.e. n=200) will be examined resulting in 400 stool samples and 800 Kato-Katz thick smears. In the entire study 1,200 stool samples will be collected and 2,400 Kato-Katz slides examined.

4.7 Outcome measurements

4.7.1. Assessment of infection performed by

1. Kato-Katz thick smears for diagnosis of hookworm (and other soil-transmitted helminth) infection in the field. Minimum acceptable samples of stool on two consecutive days per individual. 2 slides examined per stool (total 4 slides per person). Each Kato-Katz will be established and read within 30 to 45 minutes after establishment. Each parasite species will be recorded, for each species the number of eggs identified will be counted. A person is considered infected if at least one hookworm egg on 1 slide can be detected.
2. Quality control: 10% random selection of KK slides will be read by a second reader (senior microscopists).

4.7.2. Clinical assessment measurements

1) An individual questionnaire will be administered to all the participating children (interviews will be conducted by the head teachers):

- **a.** Demographics (i.e. age and sex) and socio-economic indicators (e.g. household assets ownership)
- **b.** Perceived morbidity indicators with a recall period of 2 weeks.
- **c.** Risk factors for hookworm infection (food, routine lifestyle, contact habit, standard of hygiene etc,)
- **d.** Recent medical history / other personal medical history within the last 4 weeks (including anthelminthic treatment)
- **e.** Individual side effect questionnaire will be administered
- **f.** Anthropometric measurements: height, weight, MUAC,
- **g.** Nutritional measurements: hemoglobin level

2) Individual adverse effect questionnaire form will be administered to all subjects enrolled within 24 hours following ABZ either MBZ.

4.7.3. Randomization and allocation concealment

A random numbers table will be used to generate random number sequences for the allocation of the two group treatments, created by a statistician who will not otherwise be involved in the trial.

i. Group A: single oral dose of MBZ (500-mg). Group B: single oral dose of ABZ (400-mg).
ii. Numbered containers will be used for allocation concealment.
iii. The nurse/clinician who will administer the drugs will not be same person as the one who will record the adverse events (masked to the drug treatment).
iv. The investigators (laboratory personnel, nurse/clinician administering the drugs, nurse/clinician assessing adverse events), will be blinded.
v. MBZ and ABZ will be ordered by Ministry of Health (MOH) of Lao PDR that currently have been used for mass- treatment
vi. Drugs will be packaged by a person not otherwise involved in the study in seal opaque envelopes with unique identification numbers provided by the statistician
vii. On the treatment day, drugs will be given under supervision on an empty stomach 30 minutes before meals
viii. A blinding
    a. The principle investigator and patients will see the drugs.
    b. The laboratory technicians (reading the Kato-Katz thick smears) are blinded.
    c. The nurse/clinicians who monitor the side effects are blinded.

4.8 Study procedures

The study will be carried out in one primary school in an area of known high hookworm prevalence rates (≥50%). In a baseline survey the status of hookworm infection prevalence and intensity will be assessed (other soil-transmitted helminth infections will also be assessed quantitatively). Subsequently, those children with a confirmed hookworm infection (at least 1 hookworm egg in at least one of the four Kato-Katz thick smears) will be randomly allocated either to the MBZ or the ABZ treatment group. Finally, the treatment outcome will be assessed in a follow-up survey 21-23 days after treatment.

4.8.1 Base-line procedure (Figure 1)

4.8.1.1 Inclusion criteria:

i. All schoolchildren aged 5-16 years are eligible.
ii. Infection with hookworms as confirmed by parasitological stool examination (at least 1 hookworm egg in at least one of the four Kato-Katz thick smears)
iii. Written informed consent of parents and children

4.8.1.2. Exclusion criteria:

i. Presence of any abnormal medical condition, judged by the medical doctor. If several patients experience serious adverse events the study will be stopped
ii. Recent history of anthelminthic drug treatment (albendazole, mebendazole, pyrantel, levamisole within past 4 weeks)
iii. Attending other clinical trials during the study
iv. For females: pregnancy or lactation as assessed by the medical doctor last menstrual cycle, upon initial clinical assessment
v. Children with chronic illness or acutely ill children (any disease).
vi. known or reported hypersensitivity to albendazole or mebendazole.

4.8.1.2. Information and written informed consent from parents or legal guardians of all subjects enrolled

4.8.1.3. Measurements (see Figure 1)

4.8.1.4. Randomization of subjects into 2 equal arms

4.8.1.5. Group A; single oral dose of MBZ (500-mg). Group B: single oral dose of ABZ (400-mg).

4.8.1.6 Compliance: treatment is under supervision

4.8.1.7 Patients receiving the drugs will be kept for observation for at least 3 hours for any acute adverse events (AEs). If there is any abnormal finding, the study physicians will perform full physical examination on each individual and findings will be recorded.

4.8.1.8 AEs within 24 hours of drug administration will be assessed by side effect questionnaire. Those patients who report AEs will be examined carefully by the study physician and, when necessary, action will be taken (see appendix 1).

4.8.3 Follow-up survey (21-23 days after treatment)

i. Record the number of absent children and reason to Day 21
ii. Record the number of withdrawn subject and reason to Day 21
iii. All individual receiving treatment will be revisited and all base-line measurements will be repeated using same procedures and methods (except hemoglobin concentration will not be done after treatment).
iv. All infected children will be treated at the end of the trial with PO single dose ABZ 400 mg

4.9 Analysis

All data will be double-entered into EpiData (version 3.1 Denmark). The differences between groups will be analyzed by using the Pearson X^2 test.

5. ETHICAL CONSIDERATIONS

The two study drugs which are compared in this trial have good safety profiles, are widely used and result in very few adverse events. All children enrolled in the study will benefit from treatment. All diagnosed parasitic infections will be treated according to national guidelines of Lao PDR.

The study aim and procedures will be explained to village and school authorities and health staff and their agreement obtained. Authorization to conduct the study will be obtained from the provincial and district health and education authorities. Furthermore, informed oral consent will be obtained from the provincial and district education departments as well as from the head teachers of the schools.

The study aims and procedures and benefits and possible adverse effects described to all children in Lao language before enrollment. Children can withdraw at any time. The day before the study the parents and/or legal guardians of participating children will be informed and signed the informed consent form.

Ethical approval for this study will be obtained from the Lao National Ethics Committee for Health Research (NECHR), Vientiane, and Ethical Committee of Canton of Basel-Stadt and Baselland (EKBB), Basel, Switzerland.

6. REFERENCES

Albonico M, Bickle Q, Ramsan M, Montresor A, Savioli L, Taylor M (2003). Efficacy of mebendazole and levamisole alone or in combination against intestinal nematode infections after repeated targeted mebendazole treatment in Zanzibar. Bull World Health Organ 81, 343-352.

Bethony J, Brooker S, Albonico M, Geiger SM, Loukas A, Diemert D, Hotez PJ (2006). Soil-transmitted helminth infections: ascariasis, trichuriasis, and hookworm. Lancet 367, 1521-1532.

De Clercq D, Sacko M, Behnke J, Gilbert F, Dorny P, Vercruysse J (1997). Failure of mebendazole in treatment of human hookworm infections in the southern region of Mali. Am J Trop Med Hyg 57, 25-30.

de Silva NR, Brooker S, Hotez PJ, Montresor A, Englels D, Savioli L (2003). Soil-transmitted helminth infections: upgrading the global picture. Trends Parasitol 19, 547-551.

Flohr C, Tuyen LN, Lewis S, Minh TT, Campbell J, Britton J, Williams H, Hien TT, Farrar J, Quinnell RJ (2007) Low efficacy of mebendazole against hookworm in Vietnam: two randomized controlled trials, Am J Trop Med Hyg 76, 732-736.

Katz N, Chaves A, Pellegrino J (1972). A simple device for quantitative stool thick-smear technique in schistosomiasis mansoni. Rev Inst Med Trop Sao Paulo 14, 397-400.

Keiser J, Utzinger J (2008) Efficacy of current durgs against soil-transmitted helminth infections. JAMA (in press)

Horton J (2000). Albendazole: a review of anthelmintic efficacy and safety in humans. Parasitology 121 (Suppl.), S113-132.

Hotez PJ, Bethony J, Bottazzi ME, Brooker S, Diemert D, Loukas A (2006b). New technologies for the control of human hookworm infection. Trends Parasitol 22, 327-331.

Hotez PJ, Molyneux DH, Fenwick A, Ottesen E, Ehrlich Sachs S, Sachs JD (2006a). Incorporating a rapid-impact package for neglected tropical diseases with programs for HIV/AIDS, tuberculosis, and malaria. PLoS Med 3, e102.

Utzinger J, Keiser J (2004). Schistosomiasis and soil-transmitted helminthiasis: common drug for treatment and control. Expert Opin Pharmacother 5, 263-285.

WHO (2002). Prevention and control of schistosomiasis and soil-transmitted helminthiasis: report of a WHO expert committee. WHO Tech Rep Ser No. 912.

Figure 1: Study summary on procedures and measurements taken

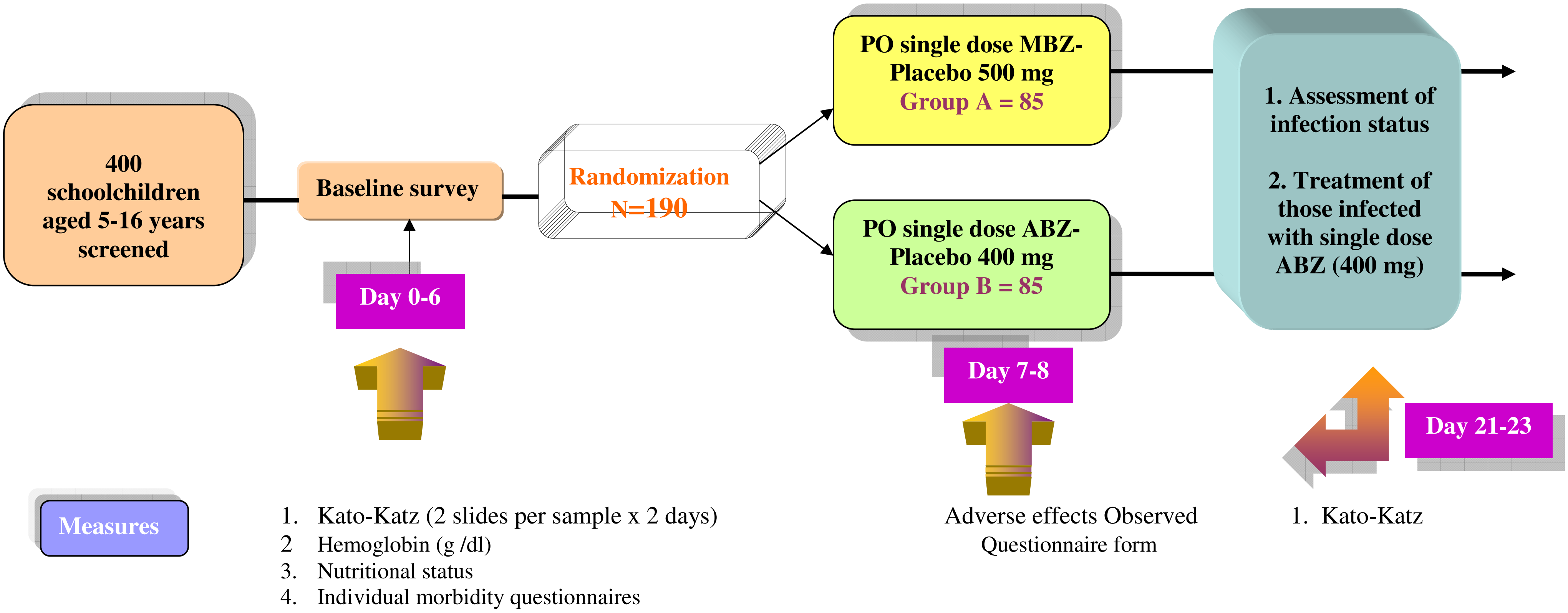

APPENDIX 1: STEP BY STEP PROCEDURES

I. Base-line survey

I.1 Preparation phase from 1st to 20th April 2008

1. Finalize research proposal with supervisor (La)
2. Submit the proposal to Lao National Ethics Committee for Health Research (NECHR) and EKBB, Basel.
3. Develop questionnaire and perform the field-test questionnaires to be piloted at least 10 individuals and modified before performing in the field. (La & research team)
4. Announce to officials and district supervisions of locations and plan of the study and require MoH and NIOPH letters and approval (Khampheng & La)
5. Order and procure of laboratory materials and other supplies (La & Somphou)

I.2 Departure to the field on 21st April 2008

i. Meet the provincial and district health officers in Champasack province
ii. Meet the director of the provincial malaria station to obtain the microscopes and recruit the technical staffs.
iii. Establish team work and plan next step.

I.3 Field collection data

On the first day:

i. Meet the head of school and obtain the list of the schoolchildren and prepare line listing (the line listings are very important to track of the enrolled subjects)
ii. Meet the parents or legal guardians and children in order to receive all the procedures and the information before enrollment
iii. Distribute informed consent form and the first stool container with clear instruction on how provide a fresh stool sample to schoolchildren with support of teachers
iv. Organize team work for following work days.

On the Second day:

i. Register and collect stool samples using X mark on the stool samples in the school Line Listing
ii. Distribute the second stool container
iii. Check for missing stool samples

On the Third day

i. Check and collect the missing second stool samples
ii. Distribute the second stool samples
iii. Check for missing stool samples
iv. Prepare for administration of drugs

On the fourth day

i. A random selection of subjects infected with hookworm infection into two equal arms performed by a research coordinator.
ii. Administration of albendazole and mebendazole in each group
iii. Treatment will be observed and elicit adverse effects at 24 h

II. Follow up at day 21-23 (from 20th to 23rd May 2008 of treatment in hookworm infection.

i. Repeat base-line measurement (except hemoglobin concentration)
ii. Treatment will be given to those with infections.

III. Measurement instruments

a. Lao consent form
b. Line listing schoolchildren
c. Individual questionnaire
d. Kato-Katz record form
e. Side effect form
f. Treatment form

APPENDIX 1: Informed Consent Form

NATIONAL INSTITUTE OF PUBLIC HEALTH
SAMSENTHAI ROAD, BAN KAO-GNOD, SISATTANAK DISTRICT,
VIENTIANE MUNICIPALITY-LAO P.D.R.
Telephone:856-21-250670, Fax: 856-21-214012.
e-mail: laoniph@laotel.com

Swiss Tropical Institute
Institut Tropical Suisse
Schweizerisches Tropeninstitut

Comparative efficacy of albendazole and mebendazole against hookworm infection: randomized, controlled trial in schoolchildren in Khamkeut district, Bolikhamxay province, Lao PDR

This research is carried out by Assoc Prof Dr Kongsap Akkhavong and Dr Phonepasong Soukhathammavong from the National Institute of Public Health, Ministry of Health, Lao PDR; by Dr Peter Odermatt and colleagues from the Swiss Tropical Institute, Basel, Switzerland.
The study is funded by the Swiss National Science Foundation and the Swiss Agency for Development and Cooperation

This informed consent form has two parts:

- **Part I is the information sheet to share information about the research study with you and your child,**
- **Part II is the certificate for your consent for signature if you agree to take part.**

You will be given a copy of the full Informed Consent Form

PART I: INFORMATION SHEET

Introduction

Soil-transmitted helminthiases in particularly hookworm infections are very common in Lao schoolchildren. Two drugs are widely used in mass-drug administrations to treat these infections: mebendazole and albendazole. There are indications that mebendazole is less efficacious to cure hookworm infections.

Purpose

The objective of this study is to comparatively assess the efficacy of oral albendazole (single 400-mg dose) and oral mebendazole (single 500-mg dose) against hookworm infection among schoolchildren in central Lao PDR.

Choice of participation: Why ask my child?

Your child has been selected by chance from all the primary and secondary schoolchildren in school.

Participation is voluntary: Does my child have to participate?

The participation is entirely voluntary. Below we will explain what the participation entails. You and your child are entirely free to decide to participate or not.

Procedures: What is going to happen to the child?
All study participants will be asked to provide 2 stool samples on two different days to the study team. The stool will be examined on worm eggs. It is important that stool samples originate from the same child. The child will be examined on the presence of intestinal parasites, if the child is infected with hookworm parasites it will be randomized to a treatment. Moreover, the child will be examined twice (the first time before the treatment and the second time). The child who is not infected with hookworm infections will not be included in the randomisation of the treatment and will not be followed-up. All infections found in this child will be treated as well. Additionally, a medical doctor will examine and interview the children on the food consumption and hygiene behaviour and a small blood sample will be taken from the finger to examine the anemia.

Risk: Will this be bad or dangerous for my child?
All the working procedures and examinations during this study are routinely conducted at a health facility. They do not bear any risks.

Discomfort: Will it hurt?
The finger-prick for blood collection with hurt a bit. However, no further procedure will hurt as none of the procedures is entering the body. However, you might be annoyed that we want to have 2 stool samples; your child might need to wait until the doctor is ready to examine and question your child.

Benefit: Is there anything good that will happen to me?
Your child will obtain all the results of his / her examination. In addition, for all the parasite infection which will be found in his/her stool examination your child will receive a free treatment. The treatment corresponds to the recommended treatment in Laos.

Incentives: Does my child get anything for the participation in the research?
Your child will not receive further incentives.

Confidentiality: Is everybody going to know about this?
All the information on your child and the results of his/her stool examination will be kept strictly confidential.

Sharing findings: Will you tell me the results?
You and your child will receive the results of all diagnostic procedures performed. Nobody except you and your child will obtain a feedback on his/her personal examination. We will also inform you and your child about the results of the study in a meeting in the school.

Right to refuse or withdraw: Do I have the right not to participate? Can I change my mind?
You and your child are absolutely free to participate and you are also free to change your mind any time.

Contact: Who can I contact to ask questions?
Dr. Phonepasong Soukhathammavong 020-248 0144 or Assoc. Prof. Dr. Kongsap Akkhavong 020-550 9725 can be conducted to ask further questions or to discuss the study.

PART II: CERTIFICATE OF CONSENT

My child has been invited to participate in this research. I understand that my child will be examined clinically by a doctor. In addition somebody will ask some questions about his/her food consumption and hygienic behaviour. My child will be asked to give two stool samples to the research team which will be examined for the presence for parasites. I understand that all this will not be painful but might be take some time and efforts from my side. Furthermore, I can count on receiving all the information on his/her examinations and receive free treatment for any parasite infection diagnosed. Furthermore, I will obtain information on the results of the study. I or my child will not receive any further compensation.

I have read all the above information, or it has been read to me. I have had the opportunity to ask questions about it and any question I asked has been answered to my satisfaction. I give the consent that my child can participate in this research study and understand that I have the right to withdraw my child from the research any time without in any way affecting his/her medical care.

Name, first name of the guardians or parent: ___________________________.

Signature: ______________________________ date: _____________________________

Location: ________________________________ Signature of witness: ______________

Signature of research interviewer: ____

APPENDIX 2: Flow chart for the work in the field site

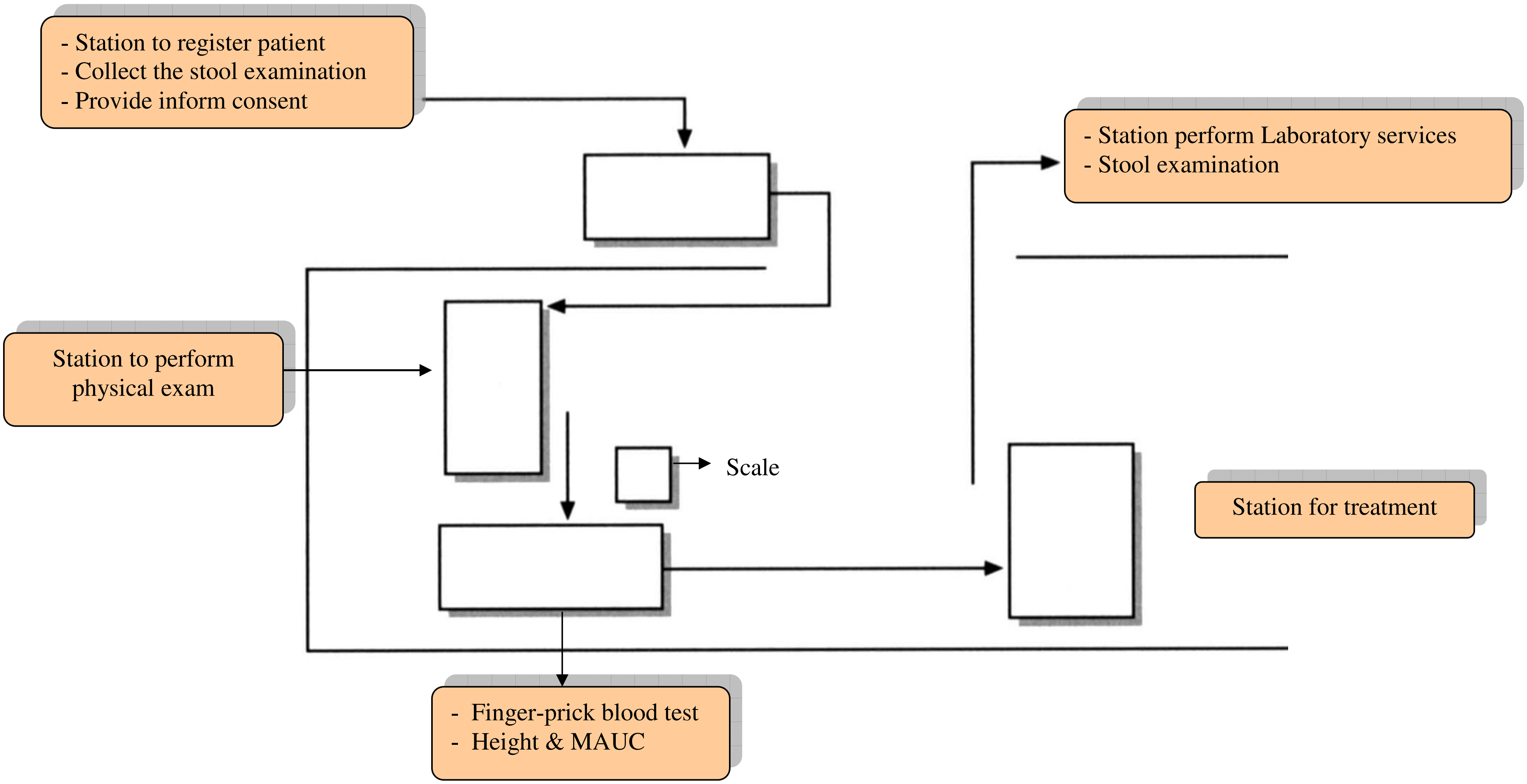

Supplement: Protocol S1 — Trial Protocol. (PDF) [file pntd.0001417.s002.pdf]
